# Food-grade filler particles as an alternative method to modify the texture and stability of myofibrillar gels

**DOI:** 10.1038/s41598-017-11711-1

**Published:** 2017-09-14

**Authors:** Andrew J. Gravelle, Shai Barbut, Alejandro G. Marangoni

**Affiliations:** Department of Food Science, University of Guelph, Guelph, ON Canada

## Abstract

A series of food grade particles were characterized for their potential as fillers in myofibrillar gels. The fillers were separated into (i) hydrophilic, insoluble, crystalline particles and (ii) starch granules. The particles used were microcrystalline cellulose, oat fiber and walnut shell flour, as well as potato and tapioca starches. Crystalline particles increased hardness and decreased recovery properties. Although all of these fillers decreased the T_2_ relaxation time of water, this was dependent on particle type and size. An increase in gel strength was observed with increasing filler content, which was attributed to particle crowding. Native potato starch was the most efficient at increasing liquid retention, while native tapioca was the least effective. Gel strength increased significantly only for the native potato and modified tapioca starches, but no effect on recovery attributes were observed for any of the starch varieties. The potato starches became swollen and hydrated to a similar extent during the protein gelation process, while the native tapioca starch gelatinized at higher temperatures, and the modified tapioca showed little evidence of swelling. T_2_ relaxometry supported this finding, as the meat batters containing native potato starch displayed two water populations, while the remaining starches displayed only a single population.

## Introduction

Finely comminuted meat products such as frankfurter-type sausages and bologna can be described as a discrete fat phase embedded in a thermally-set protein gel network^1, 2^. The chopping, or comminution process is performed under saline conditions to facilitate extraction of the salt-soluble (predominantly myofibrillar) proteins. Some of these proteins associate at the surface of the fat globules, forming an interfacial protein film (IPF), thus embedding the fat droplets within the gel matrix, as well as acting to physically restrain or stabilize the droplets during the thermal gelation process^1^. As a result, these types of products are commonly referred to as meat emulsions, or meat batters.

Comminuted meat products have a relatively high fat content (often 20–30% or greater), and as this fat generally comes from animal sources which are rich in saturated fatty acids, these products often contain a significant amount of saturates. Although there has been conflicting evidence on the role of saturated fats on human health in recent years, consumption trends have been steered towards mono- and poly-unsaturated fats^3^, and both fat- and calorie-reduced products remain popular with consumers. As a result, there have been many strategies explored for both reducing fat content and utilizing fats with more favorable lipid profiles^4, 5^. The main target of such strategies has been to decrease the caloric content or incorporate liquid vegetable oils, while maintaining product performance (e.g. yield, stability) and mimicking desirable sensory attributes, such as texture and perceived juiciness.

The addition of carbohydrates has been the dominant strategy used to retain sensory properties of comminuted meats when reducing fat content, as they can serve to improve the water holding capacity of the meat protein^6^. Starches are commonly used across the meat industry to retain moisture either by acting as bulking agents through the formation of hydrogels. Starches are a popular ingredient because they are inexpensive, and their composition and functional properties vary with plant source; the latter of which can be further tailored by various physical or chemical modifications. The use of starches as water binding agents in comminuted meats is well established^4, 6, 7^; however, various modified and underutilized sources of starch are still being actively investigated^8, 9^. Other hydrocolloids such as dietary fibres^10–12^, gums^13, 14^, and cellulose derivatives^15, 16^ have also been explored, and are still of interest in current literature.

In addition to reducing the caloric content of comminuted meats through fat reduction, strategies for achieving an improved lipid profile have represented an alternative approach to improving the nutritional profile of these products^5^. Such strategies have included using oils from plant sources which have been pre-emulsified with non-meat proteins^17, 18^, or structured via non-traditional means, including oil in water emulsions^19, 20^, gelled emulsions (i.e. the oil is dispersed in a hydrogel)^11^, or oleogelators (i.e. directly structuring the oil)^21, 22^.

Traditionally, the mechanism by which fat globules are stabilized in a comminuted meat system was thought to be via either emulsification or physical entrapment^1^. However, it was recently proposed that the capillary forces arising from the water filled channels present throughout protein network are responsible for the water holding ability of these soft materials^23, 24^, as well as stabilizing the fat phase. In a recent study, our group demonstrated that incorporating micron-sized glass beads in a lean comminuted chicken meat batter can serve to enhance the performance of the resulting composite gel by providing support to the capillary network^25^. The addition of these particles as a model insoluble hydrophilic filler dramatically decreased liquid expulsion and improved the integrity of the protein gel network; i.e. increased texture profile analysis (TPA) parameters and decreased the occurrence of microfractures induced by fluid migration. Under the capillarity hypothesis^23^, it would be expected that providing support to the capillary network would also serve to stabilize the embedded lipid droplets^24^, which could assist with reformulating comminuted meat products with improved lipid profiles. Therefore, the goal of this work was to investigate the feasibility of using food-grade hydrophilic, insoluble filler particles to serve as analogs for glass beads. The particles selected were microcrystalline cellulose (MCC, 2 sizes), oat fiber, and walnut shell flour. To contrast the crystalline particles (which are not affected by the thermal gelation process), two types of starches were investigated as fillers. Although starches are commonly used to improve liquid retention in comminuted meats, they are generally not viewed as particulate fillers, as they swell and gelatinize during thermal processing. However, if the granules maintain some crystallinity and structural integrity, it may be useful to interpret their influence on the meat protein gel in terms of particle-filled composite materials. To this end, native potato and tapioca starches were selected as fillers, as they vary in both size and gelatinization temperature. Furthermore, a modified version of each starch variety was also investigated, to determine how modifying the gelatinization temperature affected their impact on the composite meat protein gel.

## Results and Discussion

### Crystalline filler particles

#### Liquid loss and large deformation properties

**Figure 1:**
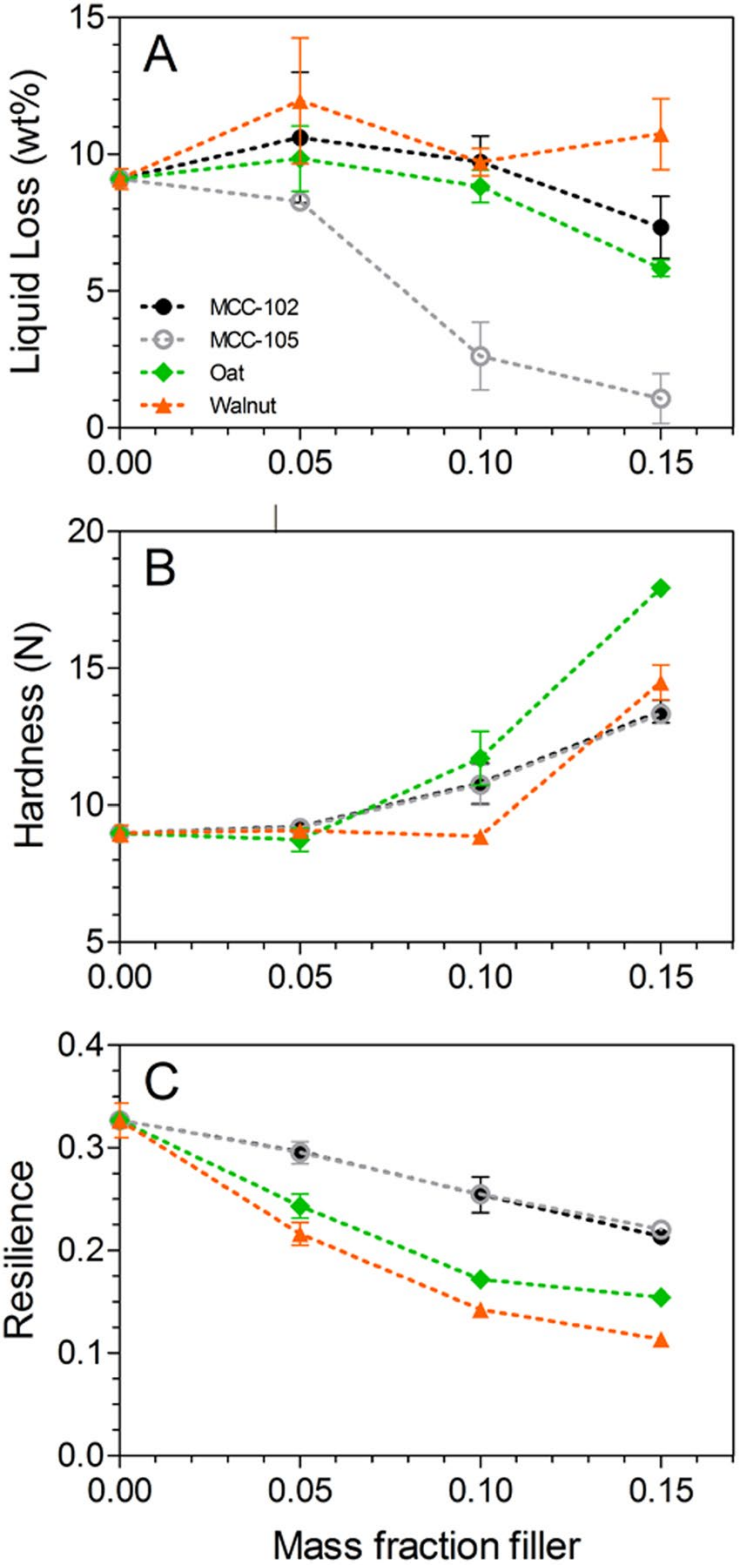
Post-gelation liquid loss (**A**), and texture profile analysis Hardness (**B**) and Resilience (**C**) of the composite meat protein gels containing crystalline particles as fillers. Fillers denoted in legend are microcrystalline cellulose (larger particles: MCC-102; smaller particles: MCC-105), oat fiber (Oat), and walnut shell flour (Walnut).

Fluid losses (fat and water) are commonly used as an indicator of stability in comminuted meat products^6, 26^, as fluid retention contributes to important attributes such as juiciness and firmness. The liquid loss during thermal gelation of the composites containing crystalline filler particles is presented in Fig. 1A, and is reported as the wt% relative to the meat batter (note: no fat loss was observed). In the absence of filler, the comminuted meat gels exuded ~9 wt% liquid. In commercial products such as frankfurters, emulsified fat binds to the protein network, acting as an ‘active’ filler, thus contributing to the stability of the protein network, and aiding in moisture retention^1, 24^. As no fat was added to the lean chicken breast meat in the present work, this level of fluid loss suggests the gel matrix was relatively stable. The addition of crystalline filler particles had little impact on further improving gel stability across the range of filler content investigated (m_*f*_ = 0–0.15). The only notable exception was MCC-105, which produced a significant decrease in liquid loss relative to the unfilled sample at m_*f*_ of 0.10 and 0.15. This result is in line with previous work published by our group using hydrophilic glass microspheres of varying sizes as model filler particles in comminuted chicken meat batters^25, 27^. Glass beads ~4 μm in diameter completely arrested liquid expulsion at low incorporation levels (volume fraction <0.05); however, the ability of these particles to stabilize the water phase rapidly diminished with increasing particle size, requiring a much higher filler content to eliminate liquid losses. Therefore,as all of the particles presented in Fig. 1 were selected for their crystalline nature and insolubility in water, the influence of the ~15 μm MCC-105 particles can likely be attributed to their smaller size (relative to the other particles), and the associated increased surface area available to interact with water. This point will be further addressed in the following subsections.

Figure 1B and C respectively depict the large deformation Hardness and Resilience, as measured by uniaxial compression to 50% of the sample’s original height. For all varieties of crystalline particles under investigation, a relatively high m_*f*_ is required to produce a significant increase in Hardness relative to the unfilled gel (Oat: m_*f*_ ≥ 0.10; MCC-102, MCC-105, Walnut: m_*f*_ = 0.15). Previous work has indicated that the textural properties of comminuted meat batters can be enhanced by stabilizing the water phase; however, an increase in composite Hardness occurred for all particle types, irrespective of their ability to decrease liquid losses. High filler loading can also result in particle-particle contacts, and when the filler particles are significantly stiffer than the surrounding matrix, such contacts will have a greater capacity to withstand deformation, thus resulting in the observed increase in Hardness^27^. Therefore, the observed increase in the strength of the composites with a high filler content can be attributed to stress-loading of the rigid particles during the deformation process as a result of filler crowding. Similar to the observed increase in gel strength here, Rayment *et al*. reported an increase in the viscosity of MCC-filled guar galactomannan solutions prepared with an increasing volume fraction filler^28^. Furthermore, Schuh *et al*. demonstrated that the firmness of full-fat comminuted meat batters gradually increased when incorporating low concentrations of MCC (0.3–2.0 wt%)^16^. In contrast, equivalent concentrations of carboxymethyl cellulose greatly decreased the firmness of these composites and drastically altered the microstructure of the protein network. These results indicate the surface chemistry of the filler particles strongly influences their compatibility with the gel network, and furthermore suggests the crystalline fillers investigated here were relatively compatible with the meat protein network (as no decrease in gel strength was observed).

The incorporation of rigid particles in a flexible matrix can contribute to stress concentration at the particle-matrix interface during deformation, which has been shown to act as a nucleation point for microfractures^29^. Such debonding from the matrix during deformation has also been shown in particle-filled food gels in which the modulus of the filler and matrix are comparable. Plucknett *et al*. characterized the debonding of spherical maltodextrin particles embedded in a continuous gelatin matrix by confocal microscopy^30, 31^, indicating weak adhesion can also contribute to fracture at the interface. At higher filler concentrations, filler-filler contacts can result in particles slipping past one another during compression, thus having a negative impact on the recovery of the composite material. Such behavior is observed in the Resilience of the composites with increasing m_*f*_ in Fig. 1C. For all particle types, the Resilience decreased with increasing m_*f*_, and this effect was more pronounced in the composites containing the walnut and oat particles. The reduced impact of the MCC particles on the Resilience may be attributed to their thin, rectangular shape. During incorporation, some of the particles would become oriented so that they either reduce stress concentration at the interface (i.e., orienting with their flat face parallel to the axis of compression), and some may align favorably with neighboring particles to reduce the occurrence of particle slips. Interestingly, despite the fact that the MCC-105 particles were able to improve the stability of the matrix (i.e. increased liquid retention), the textural properties of these composites did not show any stark differences from the other crystalline fillers.

The Resilience is defined as the ratio of work exerted by the material during the decompression of the first cycle relative to that of the compression stage; i.e. the instantaneous recovery of the material. Therefore, the observed decrease in Resilience with increasing filler content is consistent with the proposed mechanism of increasing composite Hardness at high m_*f*_ without an associated decrease in liquid loss. Furthermore, an analogous behavior to that depicted in Fig. 1C was observed for the Cohesiveness and Springiness TPA parameters, which also incorporate the recovery of the material after a second compression (see Supplementary Material, Fig. S1).

#### Microstructure

**Figure 2:**
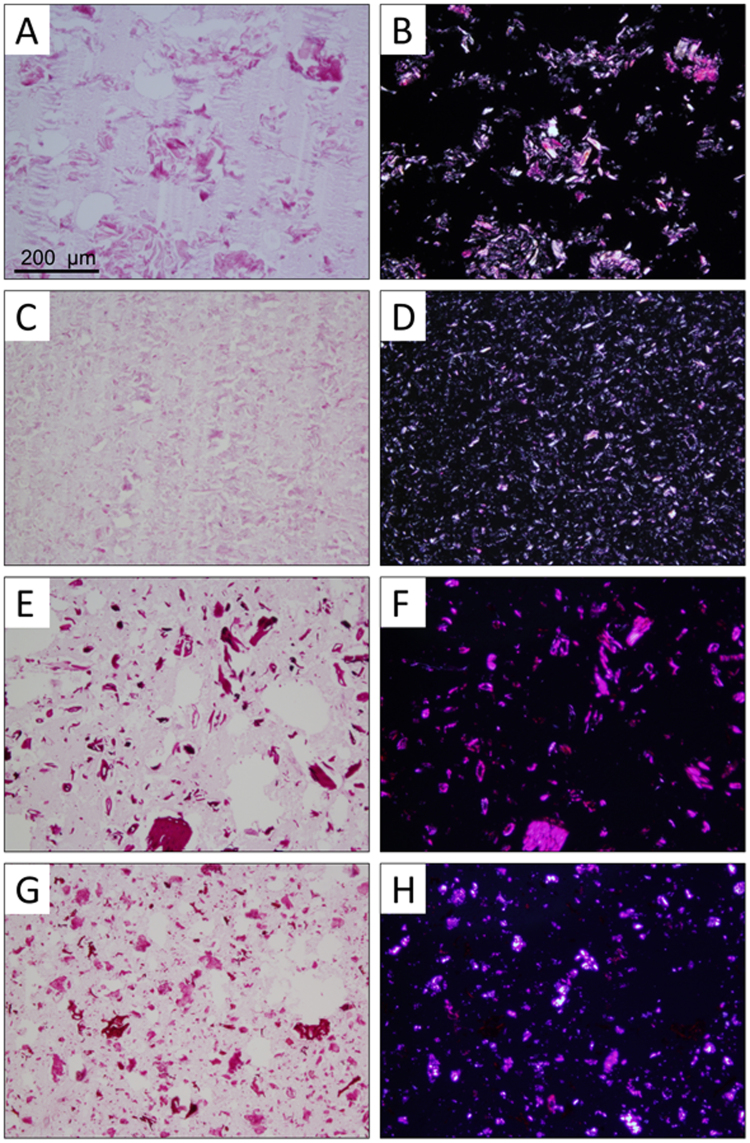
Light micrographs of composite meat protein gels containing crystalline particles as fillers (mass fraction filler, m_*f*_ = 0.10). Panels (A,C,E,G) were taken in brightfield mode, and those in Panels (B,D,F,H) were taken with polarized light. The filler particles incorporated were MCC-102 (**A**,**B**), MCC-105 (**C**,**D**), oat fiber (**E**,**F**), and walnut shell flour (**G**,**H**). All images were acquired using a 10x objective.

Light micrographs of the crystalline particle-filled meat protein gels (m_*f*_ = 0.10) are presented in Fig. 2. A PAS stain was used to highlight the carbohydrate-based filler particles (purple), and the surrounding matrix was counter-stained with H&E (pink). The left column of Fig. 2 depicts micrographs of the composites acquired in brightfield mode, while the right column depicts the same location imaged under polarized light. These images indicate the crystallinity of the particles was maintained after being subjected to the thermal gelation procedure. This was further confirmed by dispersing each class of particles in water and acquiring images both before and after mimicking the thermal gelation process (see Supplementary Material, Fig. S2). The particles appeared unaffected by the heat treatment, and no evidence of swelling was noted.

All particle types appeared to be well distributed throughout the protein matrix, and no evidence of orientation due to the preparation process was noted. It can be seen that the smaller MCC-105 particles are highly dispersed throughout the gel network relative to the other particles, which corroborates well with their ability to decrease liquid losses at higher m_*f*_. The MCC-102, walnut, and oat particles which did not provide any improvement in fluid retention, all appear to be comparable in size. This further reinforces the hypothesis that the ability of hydrophilic, insoluble filler particles to improve stability in a comminuted meat product is dictated by the available filler surface area^27, 32^.

The micrographs of the composites containing oat fiber show that the particles are particularly disconnected from the surrounding protein network (Fig. 2E). This is also seen in the composites containing walnut shell flour particles (Fig. 2G), and at higher magnification (Supplementary Material, Fig. S3), is also observed in the samples prepared with MCC-102 and even the MCC-105, albeit to a much lesser extent. Interestingly, the oat particles also produced the greatest increase in TPA Hardness (Fig. 1B) at high concentrations, and both the oat- and walnut particles had significantly lower Resilience values than those containing MCC at equivalent m_*f*_ (Fig. 1C). A similar trend was also seen in the Cohesiveness (Supplementary Material, Fig. S1B), indicating the apparent discontinuity between the particles and protein matrix is detrimental to the recovery of the composite gels.

The free space between the particles and protein matrix noted above may suggest that the myofibrillar proteins have a limited ability to adhere to these fillers during gelation, allowing free water to pool around the particles. However, as these fillers do not appear to be tightly embedded within the gel network, this interaction would not serve as an efficient means to stabilize the water phase, which is in agreement with the observed liquid losses presented in Fig. 1A. In contrast, the small size of the MCC-105 particles allows them to become more efficiently integrated into the gel network. Moreover, the available surface area increases dramatically with decreasing particle size, and it can be seen that the filler is more evenly distributed throughout the gel, resulting in a greater capacity to stabilize the liquid phase. This observation is in line with our previously published results using glass microspheres of varying sizes^27, 33^.

#### T_2_ relaxometry

**Figure 3:**
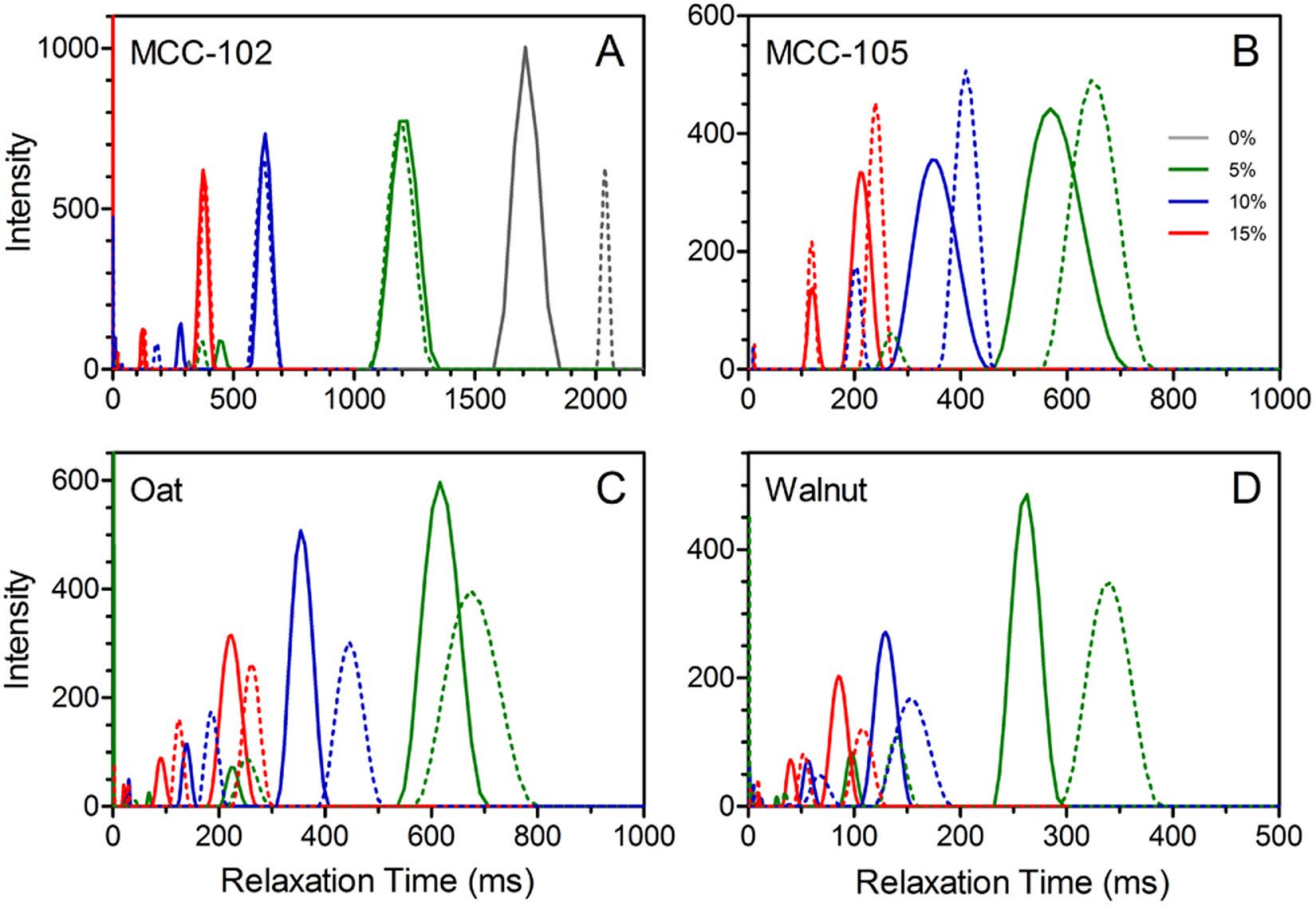
T_2_ relaxation profiles of crystalline filler particles dispersed in a 0.5 wt% xanthan gum solution prior to (solid lines) and post-thermal treatment (dashed lines); MCC-102 (**A**), MCC-105 (**B**), oat fiber (**C**), and walnut shell flour (**D**). The mass fraction filler content is denoted in the legend in Panel B. Note: Control (no filler) is only depicted in Panel A.

T_2_ relaxometry has been utilized in various meat systems to provide an indication of how the mobility of the aqueous phase is affected by various parameters, such as processing conditions and formulation changes^34–36^. Here we have investigated the effect of filler type and m_*f*_ on the relative mobility of water, both in a bulk state, and in the context of the comminuted meat batters. Figure 3 depicts the T_2_ relaxation profiles of each filler type at varying m_*f*_, and the corresponding peak relaxation times are presented in Table 1. All measurements were performed both before and after mimicking the thermal gelation procedure used to prepare the comminuted meat products. Note that all bulk water measurements were carried out using a 0.5 wt% xanthan gum solution which was used to keep the particles suspended throughout the duration of the measurement. For all filler types, the relaxation time of the dominant peak decreases with increasing m_*f*_, both pre- and post-thermal treatment. In all cases, the relaxation time increases moderately after heating. As this is also observed in the control solution (Fig. 3A, gray curves), and no discernable differences are noted in the microstructure of the particles after thermal treatment (Supplementary Material, Fig. S2), these shifts can be attributed to the effect of heating on the xanthan solution.

The T_2_ relaxation profiles presented in Fig. 3 indicate the effectiveness of the particles in decreasing the apparent water mobility were MCC-102 < MCC-105 ≈ Oat < Walnut. The relative influence of the fillers can be attributed to two factors; (i) the available surface area, and (ii) the chemical composition of the particles. Our group recently reported that the peak relaxation times of the water component in glass bead-filled comminuted meat composites are shorter when smaller particles are employed, and further decrease with increasing filler content^25, 33^. A similar effect has been reported in myofibrillar protein gels containing sugarcane fiber of varying size, where both filler size and concentration impacted the dominant T_2_ relaxation times^37^. The oat fiber and walnut shell flour should also be distinguished from the MCC, as in addition to cellulose, these particles contain other plant structuring material, such as lignin and hemicellulose. The latter is a heterogeneous carbohydrate polymer which may improve the interaction of the particles with water, due to its amorphous nature. The process by which these particles are made (i.e. grinding and milling) would also be expected to produce rougher, more pitted surfaces which would further improve their ability to interact with water. Therefore, influence of the various crystalline fillers on the peak T_2_ relaxation time of bulk water can be rationalized by the combined impact of particle size and surface properties.

It is also worth noting that the presence of xanthan gum has been shown to effect the rheological properties of particulate solutions via depletion flocculation^38^, which may decrease the apparent T_2_ of the fillers. The extent of this effect would be influenced by filler size, and any interaction between the crystalline fillers and the xanthan gum itself. Therefore, when comparing T_2_ values of the different particle types, the possibility of this effect should be kept in mind.

**Figure 4:**
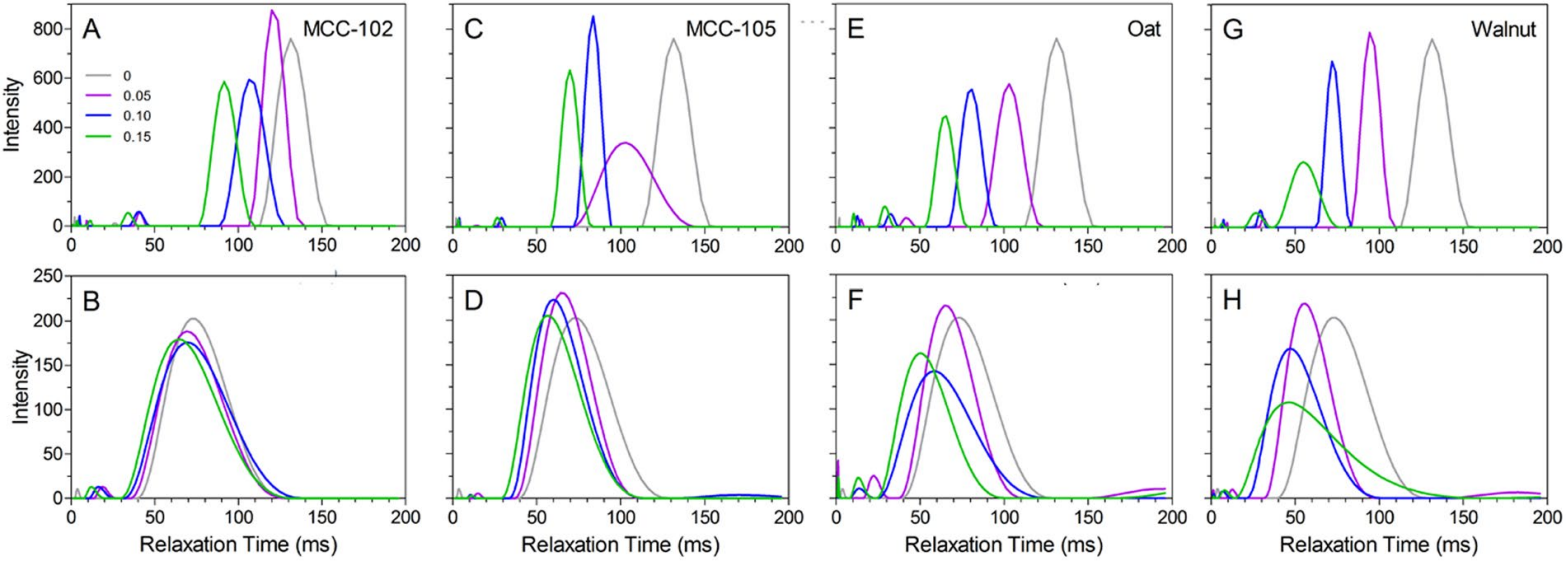
T_2_ relaxation profiles of composite meat protein gels containing various crystalline filler particles; MCC-102 (**A**,**B**), MCC-105 (**C**,D), oat fiber (**E**,**F**), and walnut flour (**G**,**H**). Relaxation profiles were acquired both prior to- (**A**,**C**,**E**,**G**) and post-thermal treatment (**B**,**D**,**F**,**H**). The mass fraction filler content is denoted in the legend in Panel A.

The T_2_ relaxation profiles of the crystalline particle-filled meat protein batters before and after thermal treatment are depicted in Fig. 4, and corresponding peak relaxation times are presented in Table 1. The relaxation profiles are typical of comminuted meat systems, with a dominant peak (denoted T_2,1_) around 50–200 ms^35, 36, 39^, which has been attributed to the water present within the comminuted protein network^34^. For all formulations, the T_2,1_ relaxation times of the composite meat systems are shorter after gelation^34, 35^, which has been attributed to the denaturation, rearrangement, and associated contraction of the protein molecules.

**Table 1:** Peak T_2_ relaxation values for the crystalline particles dispersed in a 0.5% xanthan solution, and incorporated into meat batters.

Filler	m_*f*_	T_2,1_ of particles in 0.5% xanthan		T_2,1_ of particles in meat batter	
		Pre-heating	Post-heating	Pre-heating	Post-heating
MCC-102	0	1710	2040	131	73
	0.05	1219	1200	120	69
	0.1	630	630	107	70
	0.15	357	380	100	64
MCC-105	0	1710	2040	131	73
	0.05	570	645	103	65
	0.1	350	410	83	60
	0.15	210	240	70	56
Oat	0	1710	2040	131	73
	0.05	615	675	103	65
	0.1	355	445	81	58
	0.15	220	260	65	50
Walnut	0	1710	2040	131	73
	0.05	260	340	94	55
	0.1	130	155	72	47
	0.15	85	110	55	46

Relaxation times are presented for both before (Pre-) and after (Post-) thermal treatment. All values are reported in ms.

Prior to gelation, the effect of the particles was analogous to that seen in the bulk state; T_2,1_ values decreased with increasing filler content, and the relative impact of the filler types was consistent with that reported above (i.e. MCC-102 < MCC-105 ≈ Oat < Walnut). After gelation and removal of the expelled water, there are notable differences between the filler types. The MCC-102 particles decreased the relaxation time of the composite from 68 to 62 ms; however, there was no longer an observed concentration effect. At m_*f*_ = 0.05, the smaller MCC-105 particles produced a T_2,1_ value in agreement with the MCC-102, while for those formulations which exhibited improved water retention (m_*f*_ = 0.10 and 0.15), the peak relaxation time decreased marginally to ~57 ms. For both the oat and walnut-filled gels, the T_2,1_ relaxation times decrease with increasing m_*f*_, consistent with the trend observed prior to thermal treatment. Although these particles produced composites with T_2,1_ values equivalent to, or shorter than the MCC-105-filled batters, neither produced an improvement in gel stability. This is in contrast to previous studies on glass bead-filled meat batters where a shorter T_2,1_ value in the uncooked batter was indicative of reduced water mobility. In these composites, decreasing water mobility improved liquid retention by limiting migration through the gel during heating. Furthermore, this reduced the occurrence of water channels (i.e. microfractures), thus improving the large deformation properties of the resulting composite gel^25, 33^.

Here, we propose that both filler size and the surface properties (chemical composition, surface geometry, roughness, etc.) of the particles contribute to the stability and mechanical properties of the composite gels. The ability of the MCC-105 particles to improve water retention can be attributed to their uniform small size (~15 μm) and homogeneous distribution throughout the network. The size of these particles is also comparable to that of the water channels present in finely comminuted chicken meat batters observed by SEM; ~3–4 μm, as quantified by Stevenson *et al*.^23^. Although the walnut particles are only marginally larger (~40 μm on average) and have a stronger interaction with water as evident by T_2_ relaxometry, there is a much wider size distribution, and apparent water pooling at the interface. As noted above, such pooling may be due to the chemical composition of the plant material (e.g. presence of hemicellulose), as well as a weak interaction with the protein gel matrix. The combination of these two factors diminishes the ability of these particles to restrict water mobility within the meat batter, despite the apparent decrease in T_2_ both in bulk water and in the meat batters. This effectively diminishes the ability of the walnut particles to stabilize the water within the protein network during the gelation process, rendering them inert at low m_*f*_. This interpretation is also consistent with the effect of the oat and MCC-105 particles which are both orders of magnitude larger than the capillary diameter and are less effective at reducing the T_2_ values of water in the meat system than the walnut particles.

### Starch filler particles

#### Liquid loss and large deformation properties

Starches are commonly used as fillers in processed meat products to bind water which would otherwise be exuded from the product, thus improving the textural and sensory characteristics^6^. Starch granules were selected as filler particles to contrast with the crystalline filler particles described above. Potato starch and tapioca starch were selected for their similar composition (amylose:amylopectin ratio), and distinct size differences (small and large, respectively; see Supplementary Material, Fig. S5). Additionally, for each starch variety, a modified version designed for high temperature applications was also used. For all of the starch-filled meat batters, the protein content was decreased from 10.6% (used for crystalline fillers) to 10.25% to increase liquid expulsion, thus making the impact of the particles more pronounced.

**Figure 5:**
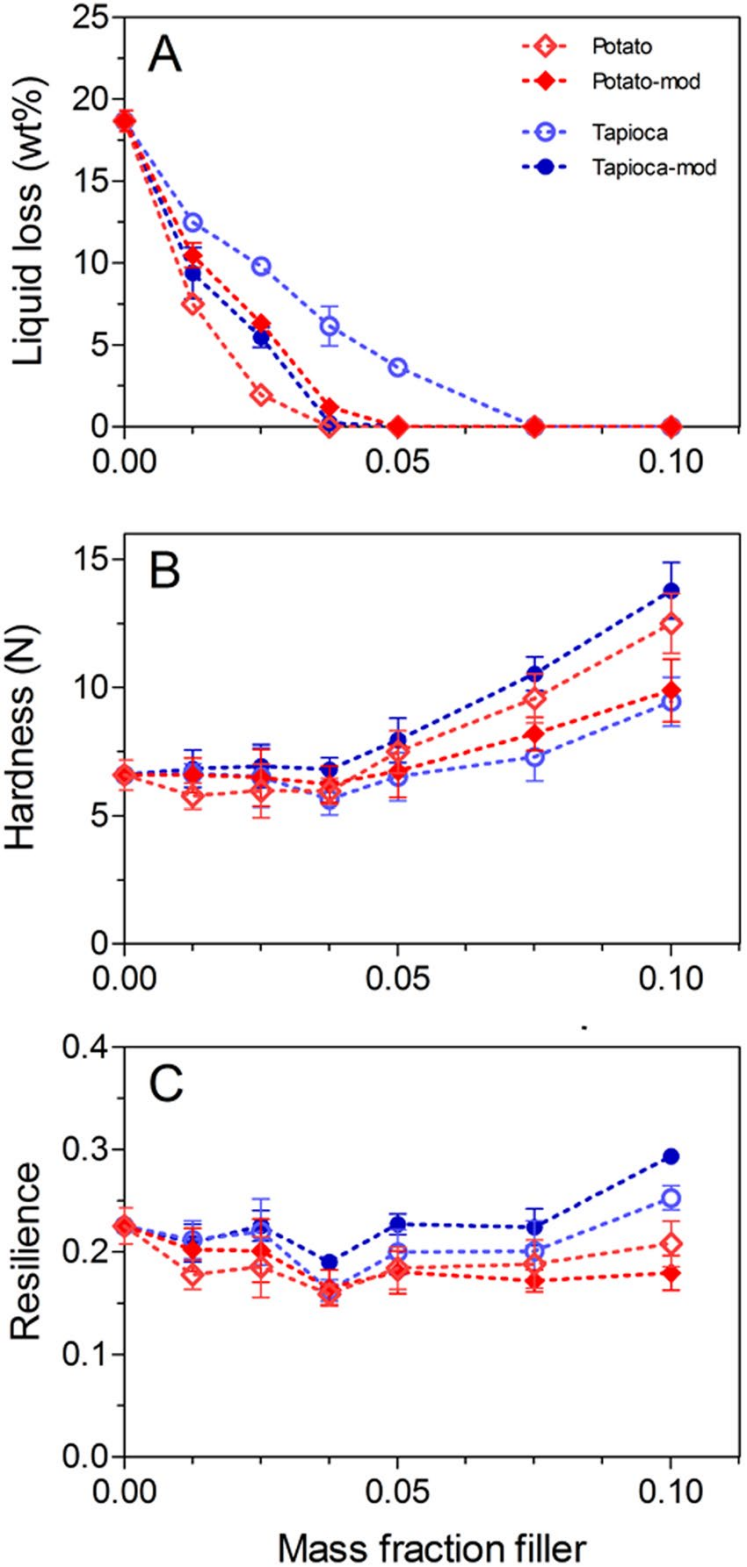
Post-gelation liquid loss (**A**), and texture profile analysis Hardness (**B**) and Resilience (**C**) of the composite meat protein gels containing potato starch (native: open diamonds; modified: filled diamonds) or tapioca starch (native: open circles; modified: filled circles).

Liquid loss of the comminuted meat batters containing various starches is presented in Fig. 5A. Overall, the decrease in liquid loss with increasing m_*f*_ occurred most rapidly with the addition of native potato starch, while native tapioca starch required higher m_*f*_ to achieve the same liquid retention. Full stabilization was achieved at m_*f*_ ≈ 0.0375 and 0.075, respectively. This may be due to the smaller granule size (decreased water holding capacity), or a higher swelling and gelatinization temperature. The latter could result in some of the mobile water being exuded from the protein network prior to the onset of swelling. The two modified starches had an equivalent, intermediate effect on improving water retention, where the modified potato starch produced a decrease in the water holding ability of the composite, while the modified tapioca showed improved liquid retention, relative to their native counterparts.

The starches had a relatively minor impact on the TPA Hardness of the composite gels (see Fig. 5B), and although there was a general increase in Hardness at higher filler content, the only samples which displayed a significant increase from the unfilled gel were those having a m_*f*_ = 0.10 of either native potato starch or modified tapioca starch. The observed increase in Hardness at high m_*f*_ can be attributed to a combination of particle crowding (outlined above), and insufficient free water available to fully hydrate the starches. The latter would result in firmer, partially swollen granules being distributed throughout the gel network, and could contribute to the Hardness of the composite material either by traditional particle reinforcement, or filler crowding. In other words, the partially swollen starch granules may behave in a manner analogous to a traditional particulate filler as opposed to forming a hydrocolloid network, which would be expected under conditions of surplus water and sufficient heating. Therefore, the significant increase from the unfilled batter seen for the native potato and modified tapioca starches at m_*f*_ = 0.10 may be attributed to the more effective water uptake/stabilization of these starches, as seen from the liquid loss (Fig. 5A). Although this argument suggests the two modified starches should have a similar impact on Hardness, their influence will also depend on the gelatinization temperature of the particular starch employed. Therefore, this discrepancy will be addressed in the following section.

An alternative interpretation of the observed increase in Hardness at high m_*f*_ may be that the excess starch particles draw additional water out of the protein network during gel formation. This would produce a more densely packed protein network, resulting in increased gel strength. However, this mechanism would effectively produce a more concentrated protein network, which should also be expected to have an effect on other textural parameters, which was not observed (Fig. 5C and Supplementary Material, Fig. S4).

The Resilience (i.e. immediate recovery) of the composite meat batters does not deviate from the unfilled control, irrespective of starch type or filler content across the entire range of m_*f*_ investigated. Although the Resilience of the composites containing the crystalline fillers gradually decreased with increasing m_*f*_, the decreased rigidity of the partially hydrated starch granules, as well as their compatibility with the protein network, resulted in an overall neutral impact. Although it was previously noted that the presence of rigid particles may lead to stress concentration at the interface^29^, the partial swelling of the granules could be expected to provide some flexibility to the filler, thus diminishing the stress concentration effect and decreasing the occurrence of microfractures at the filler/matrix interface.

#### Thermal behavior

**Figure 6:**
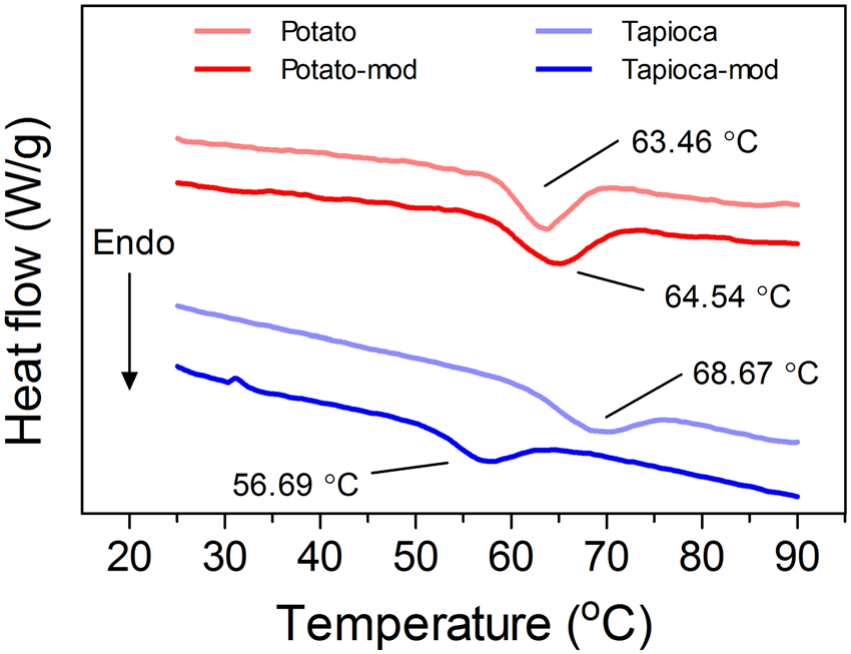
Representative DSC thermal profiles of the various starches used as filler particles in comminuted meat products. Top to bottom: native potato, modified potato, native tapioca, modified tapioca. Peak gelatinization temperature is denoted for each starch in the figure.

The thermal profiles and gelatinization temperatures (T_gel_) of the various starches used as filler particles are presented in Fig. 6. The T_gel_ of the native potato starch (63.46 ± 0.02 °C) was lower than that of native tapioca (68.67 ± 0.67 °C), which was only a few degrees below the final cooking temperature of the comminuted meat batters (72 °C). This is consistent with the observation that potato starch was more effective at increasing water retention than tapioca. Furthermore, the modified potato starch used in this study had a slightly higher T_gel_ (64.54 ± 0.11 °C), while the modified tapioca had the lowest T_gel_ of all the starches investigated (56.69 ± 0.06 °C). This is also reflected in the liquid loss of the composites, as the modified potato resulted in a significant increase in liquid expulsion than the native potato at lower m_*f*_ (0.0125–0.0375), while the modified tapioca improved liquid retention at equivalent m_*f*_. As there were no significant differences among the starches at equivalent m_*f*_ for both Hardness and Resilience, no conclusions could be drawn about the effect of T_gel_ on the textural properties of the composites. That being said, the general trend in the data suggested that within each starch variety, decreasing T_gel_ increased the TPA parameters at high m_*f*_.

Although the modified tapioca starch had the lowest peak T_gel_, native potato starch had the greatest impact on improving liquid retention. This suggests that in addition to T_gel_, differences in the structural make-up of the two starch varieties also impacts their influence on the water holding and textural properties of the starch-filled comminuted meat batters. From the present work, it appears that the T_gel_ may be used as an indicator of how a modification might impact the performance of a given starch variety (e.g. modified tapioca vs native tapioca); however, this would need to be validated with a more thorough screening of various types of modified starches, which is outside the scope of the present investigation. Furthermore, heating rates and total cooking times will also impact the extent of swelling and gelatinization, and thus the effectiveness of the starches^7, 9^.

#### Microstructure

**Figure 7:**
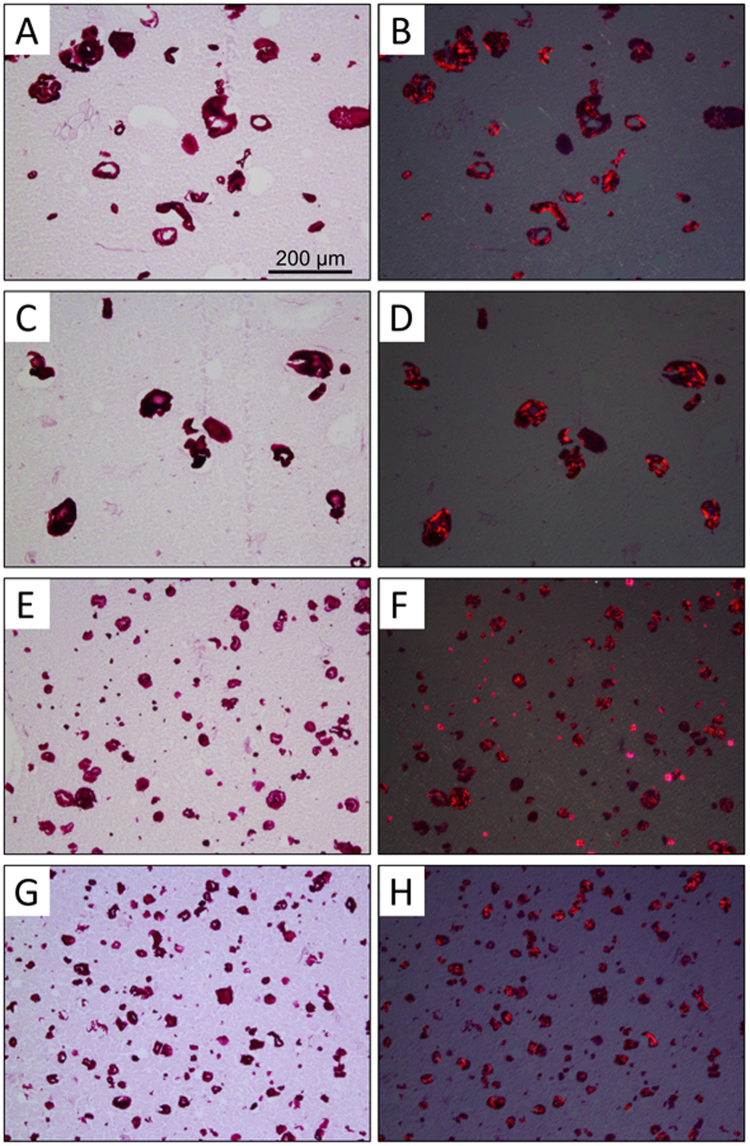
Light micrographs of composite meat protein gels containing native potato starch (**A**,**B**), modified potato starch (**C**,**D**), native tapioca starch (**E**,**F**), and modified tapioca starch (**G**,**H**) as fillers (mass fraction filler, m_*f*_ = 0.0125). Panels A, C, E, and G were acquired in brightfield mode, and corresponding images acquired with a polarizing filter are shown in Panels B, D, F, and H. All images were acquired using a 10x objective.

Micrographs of the various starch-filled comminuted meat batters (m_*f*_ = 0.0125) are presented in Fig. 7. Under polarized light, only a minor amount of crystallinity was noted, indicating the granules had undergone a significant amount of swelling, but were not completely gelatinized. Interestingly, the size of these partially hydrated starch particles were comparable to the untreated granules (see Supplementary Material, Fig. S5). By mimicking the thermal gelation process in an excess of water, it was found that the native and modified potato starch granules swelled to multiple times their original size, while the tapioca starches remained similar to that of the dry starch. Additionally, non-hydrated crystalline granules are present only in the composites containing native Tapioca (smaller particles displaying Maltese cross pattern under polarized light). This is consistent with the thermal behavior observed by DSC which indicates the peak gelatinization occurs at ~66 °C in the presence of excess water, while this shifts to ~78 °C in the meat batter (data not shown); i.e. above the final cooking temperature. Despite the differences in T_gel_, the native and modified tapioca starches appear to be swollen to a similar extent. Furthermore, when heated in excess water under conditions mimicking the meat gelation procedure, the modified tapioca starch showed little evidence of swelling, despite the absence of any crystallinity (see Supplementary Material, Fig. S5). This suggests the modification made to the tapioca starch maintained the integrity of the granule, while the lower T_gel_ implies the amylopectin (and possibly amylose) molecules undergo structural rearrangement, and partially leach from the granule during the meat batter gelation procedure.

The swelling of both the native and modified potato starch particles appears to have been physically restricted by the protein gel network, as the partially-hydrated granules are substantially smaller than when heated in excess water (Supplementary Material, Fig. S5). These images are consistent with previous studies^40, 41^, and the restricted swelling can be attributed to the fact that the myofibrillar proteins denature, aggregate, and begin to form a three-dimensional network at temperatures between 35–50 °C^2^. Although this might inhibit the ability of the starch to absorb water, the micrographs clearly indicate that the majority of the crystallinity is destroyed, indicating the particles are at least partially hydrated. As a result, at lower concentrations these particles become deformable and thus do not significantly contribute to the large deformation properties of the composite. Although more crystallinity is retained when a higher filler content is employed (due to the limited availability of water), these formulations far exceed those normally used in commercial products. Modified starches able to withstand thermal treatment and maintain their crystallinity in the final product may provide an alternative means to modify textural properties; however, as noted above, particle size is also a key factor in providing stability to comminuted meat protein gels. One would therefore require a thermally stable starch with a narrow size distribution of e.g. 1–20 μm, as beyond this size range, the ability of stiff inert particles to support the capillary network throughout the protein gel rapidly diminishes^32, 33^.

#### T_2_ relaxometry

**Figure 8:**
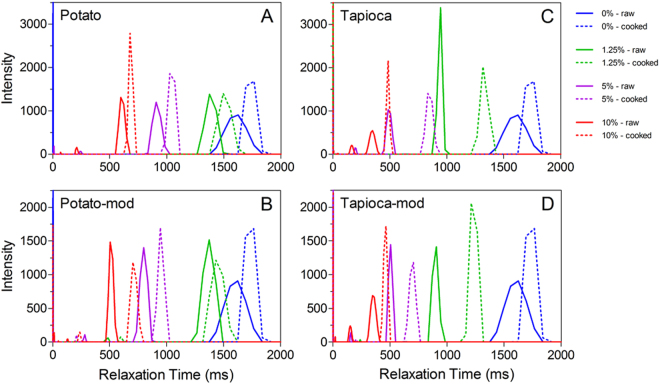
T_2_ relaxation profiles of native (**A**) and modified (**B**) potato starches, and native (**C**) and modified (**D**) tapioca starches dispersed in a 0.5 wt% xanthan gum solution prior to (solid lines) and post-thermal treatment (dashed lines). The mass fraction filler content is denoted in the legend.

T_2_ relaxation profiles of the various starch particles dispersed in xanthan gum are presented in Fig. 8. It can be seen that both the tapioca starches produced considerably shorter relaxation times than potato starch, particularly at the low to intermediate m_*f*_ tested. This difference is also more pronounced prior to thermal treatment. Consistent with the crystalline particles, the peak relaxation times increase after mimicking the thermal gelation process in excess water; however this was particularly apparent for the smaller tapioca starch granules (both native and modified). This suggests that prior to heating, the influence of the crystalline starch granules on T_2,1_ is mediated by the available surface area, as seen with the two sizes of MCC particles above. In contrast, the swollen particles are much more water-accessible, decreasing the surface-area effect, and thus the differences between the two starch varieties. Again, it should be noted that the presence of xanthan gum has been shown to cause depletion flocculation in starch dispersions^38^. It was shown this effect is more pronounced after gelatinization, due to the absorption of the water by the starch granules, effectively concentrating the xanthan in the continuous phase. Therefore, although these T_2_ values should be regarded with some skepticism, the general trend of decreasing T_2_ with increasing filler content should still be expected.

**Figure 9:**
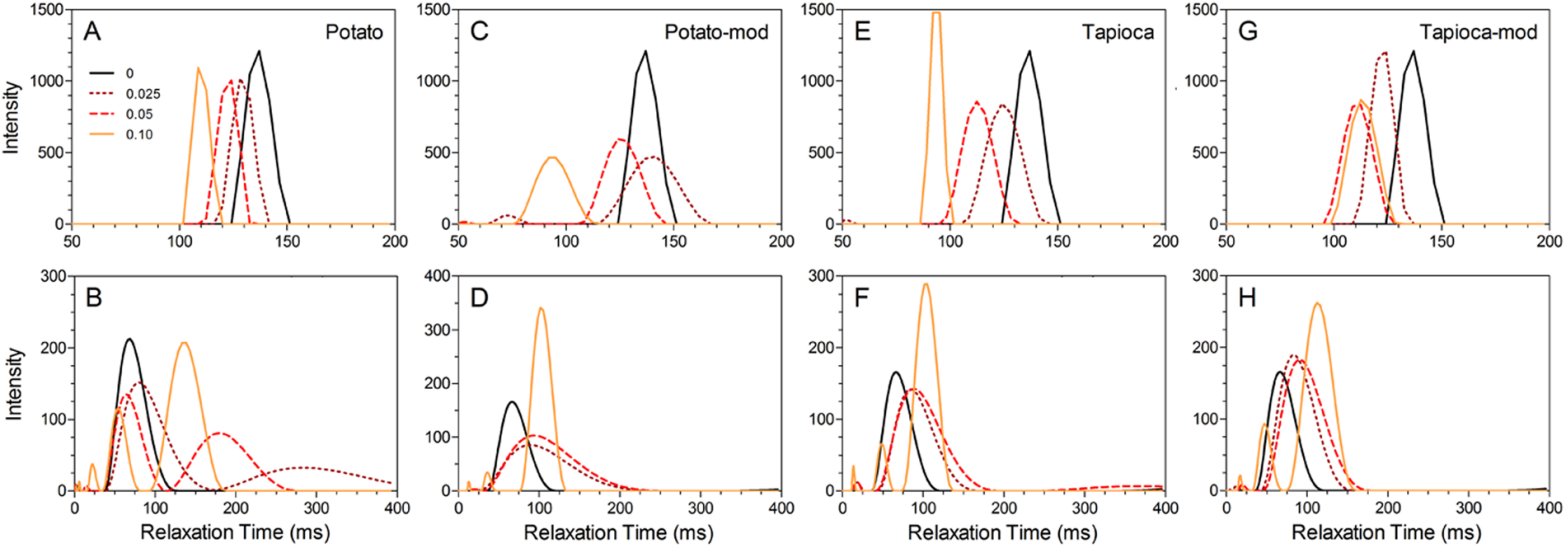
T_2_ relaxation profiles of composite meat protein gels containing native (**A**,**B**) and modified (**C**,**D**) potato starches, and native (**E**,**F**) and modified (**G**,**H**) tapioca starches. Relaxation profiles were acquired both prior to- (**A**,**C**,**E**,**G**) and post-thermal treatment (**B**,**D,F,H**). The mass fraction filler content is denoted in the legend in Panel A.

The T_2_ relaxation profiles of the starch-filled comminuted meat batters are shown in Fig. 9. Similar to that seen with the crystalline particles, prior to thermal gelation there is generally a decrease in the peak T_2,1_ relaxation time with increasing m_*f*_. After thermal processing and removal of expelled liquid, the T_2,1_ of both the unfilled batter decreased from ~135 ms to ~66 ms. Unlike the crystalline fillers which decreased T_2,1_ with increasing m_*f*_, the T_2,1_ peaks shifted to longer relaxation times (with the notable exception of native potato starch). This suggests that the water sequestered within the swollen starch granules is more mobile than that present in the protein gel network. For both the tapioca starches and the modified potato starch, a minor peak at ~48 ms was also present, indicating that when excess starch is present (i.e. there is insufficient water for hydration), two distinct water populations are observed; the faster relaxing peak associated with the water present within the protein network, and a slower relaxing population caused by the water sequestered in the partially hydrated starch granules.

The meat batters containing native potato starch appear to have a similar splitting of the T_2,1_ peak; however, for this particular starch, the split is observed at all m_*f*_ investigated, including those for which liquid losses are still observed (m_*f*_ = 0.025). As m_*f*_ was increased, the T_2_ value of both populations decreased, indicating the starch granules have a less open structure due to the shortage of water available for hydration, and thus also marginally decreasing the relaxation time of the water population associated with the protein matrix.

From these results, we conclude that pulsed NMR relaxometry may prove to be an interesting tool to probe the mechanism of water binding and how filler-matrix interactions influence the efficiency of fillers or binders in comminuted meat products. Furthermore, the partially hydrated starch granules seem to behave more akin to soft filler particles than an effective water binding agent, due to either their higher gelatinization temperature or chemical modification. It would thus be expected that increasing the gelatinization temperature of the granules would cause them to further maintain their crystalline structure, thus allowing them to behave as a rigid filler particle. The effect of the filler would then be mediated by the filler modulus and available surface area^42^. Conversely, water binding might be improved by decreasing the gelatinization temperature via processing and/or by selecting a starch with a naturally lower gelatinization point.

## Conclusion

In the present study, we have attempted to expand on previous work showing glass microspheres can improve the stability and large deformation properties of comminuted meat protein gels, with a size, or surface area dependence. To this end, a series of food-grade fillers were investigated, and separated into two categories; (i) hydrophilic, insoluble, crystalline particles, and (ii) thermally sensitive starch granules. Of the crystalline fillers characterized, only the ~15 μm MCC particles improved the liquid retention of the composite meat gels, suggesting the effect is surface area-mediated. Textural properties were only affected at high filler content (m_*f*_ ≥ 0.10), where all particles produced an increase in gel strength, and an associated decrease in recovery properties, indicated the effect was due to particle crowding. Microstructure confirmed the particles were loosely associated with the surrounding matrix, as evident by discontinuities at the filler/gel interface. T_2_ relaxometry of the raw batters indicated the water was less mobile with increasing filler content; however, this did not necessarily translate to a more stable product. It was found that particle size was the dominant factor in determining the influence of the filler on stability. It was proposed that hydrophilic fillers are most effective at improving water retention when they are comparable in size to the capillary channels present throughout the porous gel matrix.

Of the four starches investigated, it was found that the native potato starch was more effective at improving water retention at lower m_*f*_ than native tapioca, and can be attributed to its larger size and lower T_gel_. Thermal analysis indicated the modified potato and tapioca starches had a higher and lower T_gel_, which was correlated to a decrease and increase in liquid retention, respectively. The native potato and modified tapioca starches also produced harder composite gels at the highest filler content investigated (m_*f*_ = 0.10); however, the recovery properties were not influenced by the addition of any starch variety. Microstructural analysis indicated the potato starch granules were more swollen than the tapioca, but this swelling may have been restricted by the formation of the protein gel network.

T_2_ relaxometry indicated that the native potato starch more readily hydrated in the comminuted meat batter, resulting in decreased liquid losses, and the appearance of two distinct water populations; a faster population associated with the water within the protein network, and a slower population resulting from the water within the partially hydrated/gelatinized starch. In contrast, the three remaining starches only exhibited this splitting of populations at high m_*f*_, when insufficient water was available for hydration. These results suggested that the higher gelatinization temperature of native tapioca starch and chemical modifications of both the modified starches causes them to behave akin to the hydrophilic crystalline filler particles; however, the partial gelatinization of the granules resulted in higher liquid retention and no observed decrease in the recovery properties of the composite gels. Therefore, although starches are commonly used to promote water binding by forming a hydrocolloid network, under the processing conditions used here, the influence of the two starch varieties on the comminuted meat batters were similar to that of a particle-filled network. Overall, insoluble hydrophilic fillers showed potential as a means to improve the stability of comminuted meat gels when the particles are of the same order of magnitude as the capillary channels present throughout the protein network. As the starches were larger and less rigid after undergoing thermal treatment, they did not show the same potential to serve as a particulate filler in meat batters (i.e. by supporting the capillary network). Further work should be carried out to determine if this strategy could be applied to other polymer hydrogel systems.

## Materials and Methods

### Materials

Fresh boneless, skinless chicken breast meat (~25 kg) was purchased from a national supermarket (Kirkland Signature, Costco Wholesale Canada Ltd., Ottawa, ON, Canada). Within 24 hrs of purchasing, all visible fat and connective tissue was removed and the meat was chopped in a bowl chopper at the low speed setting for approximately 60 sec and mixed by hand to produce a homogeneous batch. The meat was then portioned into ~500 g batches in bags, vacuum-packed, and stored at −20 °C until use. Protein content was determined, in triplicate, to be 20.5 wt% using the Dumas method and a nitrogen conversion factor of 5.53 was employed^43^. The pH of the chicken was determined by thoroughly mixing 5 g of defrosted meat in 45 g deionized water, and the solution was then strained through glass wool prior to analysis. The pH of the liquid was measured to be 5.85 using a benchtop pH meter.

Two classes of fillers were investigated; (i) insoluble food-grade fillers, predominantly composed of crystalline material, and (ii) hydratable starches, both native and modified. The latter group will be denoted as ‘starches’, while the former class will be referred to as ‘crystalline fillers,’ as they maintain their crystallinity through the heating/gelation process (see Supplementary Material; Fig. S2). Microcrystalline cellulose (MCC) was obtained from JRS Pharma (Patterson, NY, USA) in two size ranges; VIVAPUR® 102 and 105 (average particle size of ~130 μm and 15 μm, respectively, from manufacturer specifications). Walnut flour (“walnut”; mesh size 325) and oat fiber (“oat”; Canadian Harvest) were received from EcoShell Inc. (Corning, CA, USA) and LV Lomas (Brampton, ON, Canada), respectively. Native tapioca starch, modified tapioca starch (PURITY® 87), and modified potato starch (PenBind® 140) were received from Ingredion Canada (Mississauga, ON, Canada). Native potato starch was obtained from Hela Spice Canada Inc. (Uxbridge, ON, Canada). The two modified starches used were developed for high temperature applications; however, the nature of the chemical modification is proprietary information, and these starches are thus simply denoted as “modified”.

### Preparation of particle-filled meat protein gels

All composite meat gels were prepared in a household food processor. Batters containing crystalline particles were formulated to have a final protein content of 10.6% in the batter (i.e. the gel phase), while the starch-filled gels had a protein content of 10.25%. The batters were formulated independent of the m_*f*_ employed so that the protein content of the gel matrix was constant for each class of particles. This was done to ensure the physical properties of the continuous phase in the composite was consistent across all m_*f*_ tested. Filler particles were added on a wt% or mass fraction basis; for the crystalline particles, a filler content of 0, 5, 10, and 15% was used, while for the starches, the meat batters were prepared with 0, 1.25, 2.5, 3.75, 5.0, 7.5, and 10.0% filler. All fillers were added after the chopping procedure to maintain the original size distribution, and were incorporated by hand mixing. Prior to preparation, the meat was completely defrosted overnight under refrigerated conditions (~4 °C). The meat batters were prepared by first chopping two parts meat for 60 sec, followed by the addition of one part deionized water (10 sec chopping), and 2.5% NaCl (10 sec chopping). The slurry was then held in an ice bath for 5 min to allow for the extraction of salt-soluble myofibrillar proteins. The ionic strength of the mixture during extraction was ~0.42 M. After extraction, the remaining water was added and the mixture was further chopped for a total of 80 sec. To ensure the batter was chopped homogeneously, the walls and base of the food processor were scraped at regular intervals throughout the preparation procedure. After chopping, the particles were thoroughly mixed into the batter by hand (~2 min), and each sample was equilibrated under refrigeration conditions (~4 °C) for a minimum of 1 hr prior to thermal processing. All formulations were independently prepared, and each was repeated three times in a randomized block design.

After chilling, for each composite batter, 40 g samples were stuffed into four 50 ml polypropylene centrifuge tubes and centrifuged at a low speed for 30 sec to remove air pockets. The composite batters were cooked by gradually heating to an internal temperature of 72 °C in a water bath. The heating process took approximately 75 min and the core temperature was monitored using a thermocouple unit fed through a rubber stopper. Upon reaching the target temperature, the samples were transferred to an ice bath to arrest the cooking/gelation process. Once the core temperature was below 40 °C, liquid loss was determined (see below), and the samples were then refrigerated overnight prior to performing texture profile analysis.

### Liquid Loss

After the initial cooling (<40 °C), the composite gels were equilibrated to room temperature and the excess liquid which was expelled during thermal treatment was drained and weighed. Liquid loss was expressed as the mass of the total expelled liquid relative to the mass of the meat batter (i.e. excluding the filler) prior to thermal treatment. Due to the low fat content of the chicken breast meat, no fat loss was observed.

### Texture profile analysis (TPA)

Evaluation of mechanical and textural properties of the gels was carried out using a two cycle uniaxial compression test^44^. For each sample, a total of 12 cylindrical cores (height: 10 mm; diameter: 15 mm) were compressed twice between two parallel plates to 50% of their original height using a texture analyzer (model TA.XT2, Stable Micro Systems, Texture Technologies Corp., Scarsdale, NY, USA) outfitted with a 30 kg load cell. The crosshead speed was fixed at 1.5 mm/s and all composites were tested at room temperature. From this test, a number of parameters were obtained, including Hardness, Resilience, Springiness, and Cohesiveness^44^.

### Differential Scanning Calorimetry (DSC)

The gelatinization temperature of the various starches was evaluated using a DSC 1 instrument (Mettler-Toledo, Mississauga, ON, Canada). Approximately 1 mg of starch powder was placed into an aluminum DSC pan, and deionized water was added to produce a ~10 mg 10% starch solution. The pan was then hermetically sealed and heated from 20–90 °C at a rate of 5 °C/min. Peak integration was determined using the Star Software provided with the DSC unit.

### Light microscopy

The particle-filled myofibrillar protein gels were prepared for light microscopy following a procedure described previously^45^. Briefly, ~3 mm thick discs were sectioned, encased in a cassette, fixed in formalin, dehydrated in a series of alcohols, embedded in a paraffin block. The blocks were then sectioned in ~7–10 μm thick slices, deparaffinised, and stained with Periodic acid-Schiff (PAS), using hematoxylin and eosin as a counter-stain. Samples were imaged on an optical microscope (model BX60, Olympus Optical Co., Ltd., Japan) and images were captured with a digital camera using the cellSens software (v1.0, Olympus Optical Co., Ltd.). Image analysis was carried out using Image-Pro Premier v9.1 (Media Cybernetics, Inc., Rockville, MD, USA). Images of the particles were also obtained, both immediately after being dispersed in water, as well as after a 24 hr soaking period. Lastly, effect of heat treatment on particle morphology was determined by subjecting the dispersed particles to the same thermal processing conditions used for the gelation procedure. Particles were imaged by pipetting ~20 μl of solution onto a glass microscope slide and covering with a glass coverslip.

### Low-field pulsed NMR spectroscopy

T_2_ relaxation values were obtained for the composites, both prior to and post-gelation. The effect of the particles on the relaxation time of bulk water was also determined by dispersing the particles in a 0.5 wt% xanthan gum solution, which was used to ensure the particles remained suspended throughout the duration of the experiment. Measurements were carried out on a 20 MHz (0.47 T) mq 20 series bench-top NMR spectrometer (Bruker Corp., Milton, ON, Canada), with the sample chamber maintained at room temperature (23 °C). The free induction decay was acquired from a Carr-Purcell-Meiboom-Gill (CPMG) spin echo pulse train^46, 47^, using 32 scan repetitions. The 90° and 180° pulse lengths were optimized using an automated calibration procedure, with characteristic values of approximately 2.6 μs and 6.2 μs, respectively. The pulse delay τ was set to 150 μs when analyzing the particle-filled batters, and 500 μs for the particles dispersed in xanthan. Raw meat batters and particle dispersions were transferred into small, disposable glass NMR tubes (height: 40 mm; diameter: 7 mm). A free induction decay (FID) was collected prior to thermal treatment, and the samples were then heated for 10 min in a water bath maintained at 72 °C and subsequently acclimated to room temperature in a water bath at 23 °C. For the gelled meat batters, water which was expelled during heating was decanted prior to collecting an additional FID of the thermally treated sample. No separation/sedimentation was observed in the particle dispersions. T_2_ relaxation profiles were obtained by processing the FID data with the CONTIN algorithm (Bruker Corp.) which extracts multiple rate constants using an inverse Laplace transform. The peak relaxation times were extracted using the PeakFit software package (v4.12, Systat Software Inc., San Jose, CA, USA).

### Graph plotting and statistical analysis

Data analysis and graph plotting was performed using GraphPad Prism 5 (GraphPad Software, Inc., San Diego, CA, USA). Statistical analysis was carried out within each class of particles (crystalline or starch) using a 1-way ANOVA with a Tukey post-test.

### Data availability

All data are freely available upon request to Alejandro Marangoni (amarango@uoguelph.ca) and will be supplied in Excel sheets for inspection.

## Electronic supplementary material

Supplementary Information

## References

[1] Gordon A, Barbut S (1992). Mechanisms of Meat Batter Stabilization: A Review. Crit. Rev. Food Sci. Nutr..

[2] Tornberg E (2005). Effects of heat on meat proteins – Implications on structure and quality of meat products. Meat Sci..

[3] WHO. Global Strategy on Diet, Physical Activity and Health. *World Heal. Organ*. **2002** (2004). 10.1177/156482650402500310 15460274

[4] Jiménez-Colmenero F (1996). Technologies for developing low-fat meat products. Trends Food Sci. Technol..

[5] Jiménez-Colmenero F (2007). Healthier lipid formulation approaches in meat-based functional foods. Technological options for replacement of meat fats by non-meat fats. Trends Food Sci. Technol..

[6] Barbut, S. In *The science of poultry and meat processing* (http://www.poultryandmeatprocessing.com/, at http://www.poultryandmeatprocessing.com/ (2015).

[7] Li JY, Yeh AI (2003). Effects of starch properties on rheological characteristics of starch/meat complexes. J. Food Eng..

[8] Das SK, Prabhakaran P, Tanwar VK, Biswas S (2015). Effect of some plant starches and carrageenan as fat substitutes in chicken patties. J. Anim. Sci..

[9] Kong W (2016). Effects of modified starches on the gel properties of Alaska Pollock surimi subjected to different temperature treatments. Food Hydrocoll..

[10] Choi YS (2014). Physicochemical properties and sensory characteristics of reduced-fat frankfurters with pork back fat replaced by dietary fiber extracted from makgeolli lees. Meat Sci..

[11] Herrero AM, Ruiz-Capillas C, Pintado T, Carmona P, Jimenez-Colmenero F (2016). Infrared spectroscopy used to determine effects of chia and olive oil incorporation strategies on lipid structure of reduced-fat frankfurters. Food Chem..

[12] Tomaschunas M (2013). Changes in sensory properties and consumer acceptance of reduced fat pork Lyon-style and liver sausages containing inulin and citrus fiber as fat replacers. Meat Sci..

[13] Jiménez-Colmenero F (2010). Technological and sensory characteristics of reduced/low-fat, low-salt frankfurters as affected by the addition of konjac and seaweed. Meat Sci..

[14] Salcedo-Sandoval L, Cofrades S, Ruiz-Capillas Pérez C, Solas MT, Jiménez-Colmenero F (2013). Healthier oils stabilized in konjac matrix as fat replacers in n-3 PUFA enriched frankfurters. Meat Sci..

[15] Schmiele M, Nucci Mascarenhas MCC, da Silva Barretto AC, Rodrigues Pollonio MA (2015). Dietary fiber as fat substitute in emulsified and cooked meat model system. LWT - Food Sci. Technol..

[16] Schuh V (2013). Impact of carboxymethyl cellulose (CMC) and microcrystalline cellulose (MCC) on functional characteristics of emulsified sausages. Meat Sci..

[17] Youssef MK, Barbut S (2011). Fat reduction in comminuted meat products-effects of beef fat, regular and pre-emulsified canola oil. Meat Sci..

[18] Kang ZL, Chen FS, Ma HJ (2016). Effect of pre-emulsified soy oil with soy protein isolate in frankfurters: A physical-chemical and Raman spectroscopy study. LWT - Food Sci. Technol..

[19] Herrero AM, Carmona P, Pintado T, Jiménez-Colmenero F, Ruiz-Capillas C (2012). Lipid and protein structure analysis of frankfurters formulated with olive oil-in-water emulsion as animal fat replacer. Food Chem..

[20] López-López I, Cofrades S, Yakan A, Solas MT, Jiménez-Colmenero F (2010). Frozen storage characteristics of low-salt and low-fat beef patties as affected by Wakame addition and replacing pork backfat with olive oil-in-water emulsion. Food Res. Int..

[21] Barbut S, Wood J, Marangoni A (2016). Potential use of organogels to replace animal fat in comminuted meat products. Meat Sci..

[22] Kouzounis D, Lazaridou A, Katsanidis E (2017). Partial replacement of animal fat by oleogels structured with monoglycerides and phytosterols in frankfurter sausages. Meat Sci..

[23] Stevenson CD, Dykstra MJ, Lanier TC (2013). Capillary pressure as related to water holding in polyacrylamide and chicken protein gels. J. Food Sci..

[24] Liu W, Lanier TC, Osborne JA (2016). Capillarity proposed as the predominant mechanism of water and fat stabilization in cooked comminuted meat batters. Meat Sci..

[25] Gravelle AJ, Marangoni AG, Barbut S (2016). Insight into the mechanism of myofibrillar protein gel stability: Influencing texture and microstructure using a model hydrophilic filler. Food Hydrocoll..

[26] Jongberg S, Terkelsen LDS, Miklos R, Lund MN (2015). Green tea extract impairs meat emulsion properties by disturbing protein disulfide cross-linking. Meat Sci..

[27] Gravelle, A. J., Marangoni, A. G. & Barbut, S. The influence of particle size and protein content in particle-filled myofibrillar protein gels. *Meat Muscle Biol*. In press (2017).

[28] Rayment P, Ross-Murphy SB, Ellis PR (2000). Effect of size and shape of particulate inclusions on the rheology of guar galactomannan solutions. Carbohydr. Polym..

[29] Gao YC, Lelievre J (1994). A theoretical analysis of the strength of composite gels with rigid filler particles. Polym. Eng. Sci..

[30] Plucknett KP, Normand V, Pomfret SJ, Ferdinando D (2000). ‘Ductile’ mixed biopolymer gel composites. Polymer.

[31] Plucknett KP (2001). Dynamic experimentation on the confocal laser scanning microscope: Application to soft-solid, composite food materials. J. Microsc..

[32] Gravelle AJ, Barbut S, Marangoni AG (2015). Influence of particle size and interfacial interactions on the physical and mechanical properties of particle-filled myofibrillar protein gels. RSC Adv..

[33] Gravelle AJ, Marangoni AG, Barbut S (2017). Filled myofibrillar protein gels: Improving cooking loss and texture with model filler particles. Food Struct..

[34] Bertram HC (2001). Origin of multiexponential T2 relaxation in muscle myowater. J. Agric. Food Chem..

[35] Bertram HC, Kristensen M, Andersen HJ (2004). Functionality of myofibrillar proteins as affected by pH, ionic strength and heat treatment – a low-field NMR study. Meat Sci..

[36] Stevenson CD, Liu W, Lanier TC (2012). Rapid Heating of Alaska Pollock and Chicken Breast Myofibrillar Protein Gels as Affecting Water-Holding Properties. J. Agric. Food Chem..

[37] Zhuang X (2016). Influence of sugarcane dietary fiber on water states and microstructure of myofibrillar protein gels. Food Hydrocoll..

[38] Abdulmola NA, Hember MWN, Richardson RK, Morris ER (1996). Effect of xanthan on the small-deformation rheology of crosslinked and uncrosslinked waxy maize starch. Carbohydr. Polym..

[39] Yang H (2016). Effect of protein structure on water and fat distribution during meat gelling. Food Chem..

[40] Comer FW, Allan-Wojtas P (1988). Functional and microstructural effects of fillers in comminuted meat products. Food Struct..

[41] Zhang L, Barbut S (2005). Effects of regular and modified starches on cooked pale, soft, and exudative; normal; and dry, firm, and dark breast meat batters. Poult. Sci..

[42] Ahmed S, Jones FR (1990). A review of particulate reinforcement theories for polymer composites. J. Mater. Sci..

[43] Mariotti F, Tomé D, Mirand PP (2008). Converting nitrogen into protein–beyond 6.25 and Jones’ factors. Crit. Rev. Food Sci. Nutr..

[44] Bourne MC (1978). Texture profile analysis. Food Technol..

[45] Youssef MK, Barbut S (2009). Effects of protein level and fat/oil on emulsion stability, texture, microstructure and color of meat batters. Meat Sci..

[46] Carr HY, Purcell EM (1954). Effects of diffusion on Free precession in nuclear magnetic resonance experiments. Phys. Rev..

[47] Meiboom S, Gill D (1958). Modified spin‐echo method for measuring nuclear Relaxation times. Rev. Sci. Instrum..

