# Supplementary material for: Food-grade filler particles as an alternative method to modify the texture and stability of myofibrillar gels
Source: Sci Rep. 2017 Sep 14;7:11544. doi: 10.1038/s41598-017-11711-1 (PMC5599672; doi:10.1038/s41598-017-11711-1)
Supplement: Supplementary file 1 — Supplementary Information [file 41598_2017_11711_MOESM1_ESM.pdf]

Food-grade filler particles as an alternative method to modify the texture and stability of myofibrillar gels

Andrew J. Gravelle, Shai Barbut, & Alejandro G. Marangoni*

Department of Food Science, University of Guelph, Guelph, ON, Canada

*E-mail: amarango@uoguelph.ca

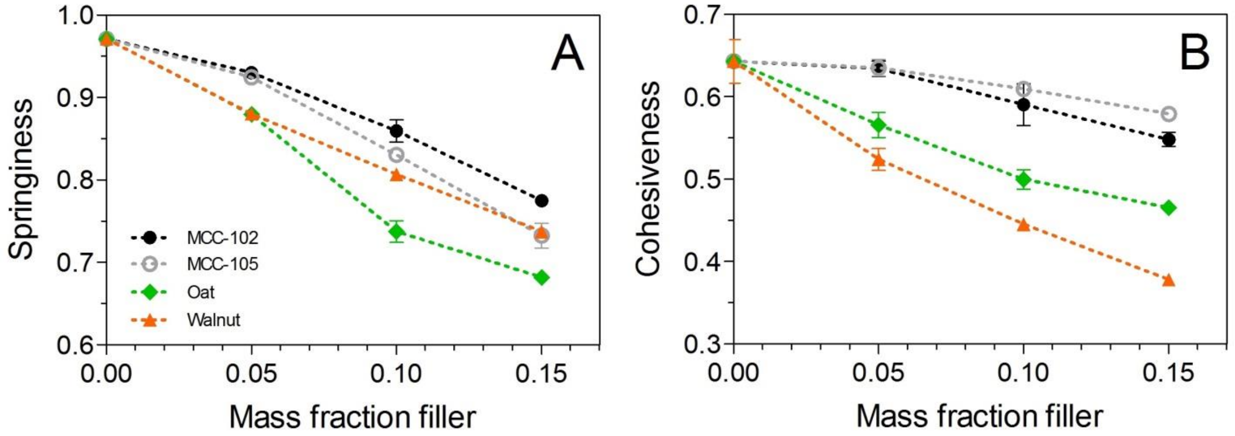

Fig. S1 Texture profile analysis (TPA) Springiness (A) and Cohesiveness (B) of comminuted meat protein gels containing crystalline particles as fillers. Results are presented as a function of mass fraction filler.

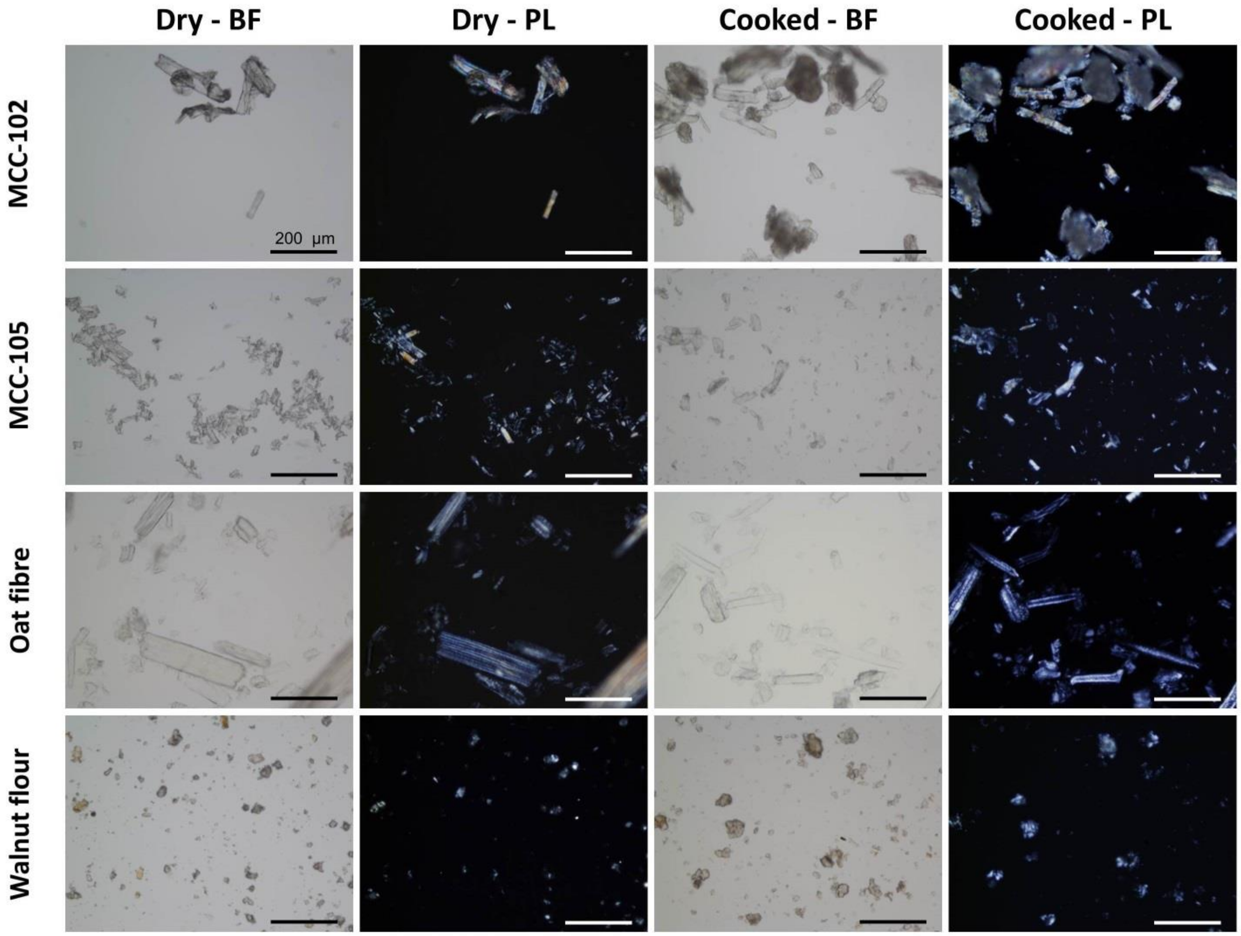

Fig. S2 Micrographs of crystalline particles used as fillers in comminuted meat batters, before and after thermal gelation (denoted “Dry” and “Cooked”, respectively). BF indicates brightfield, and PL indicates polarized light. All images were acquired with a 10x objective.

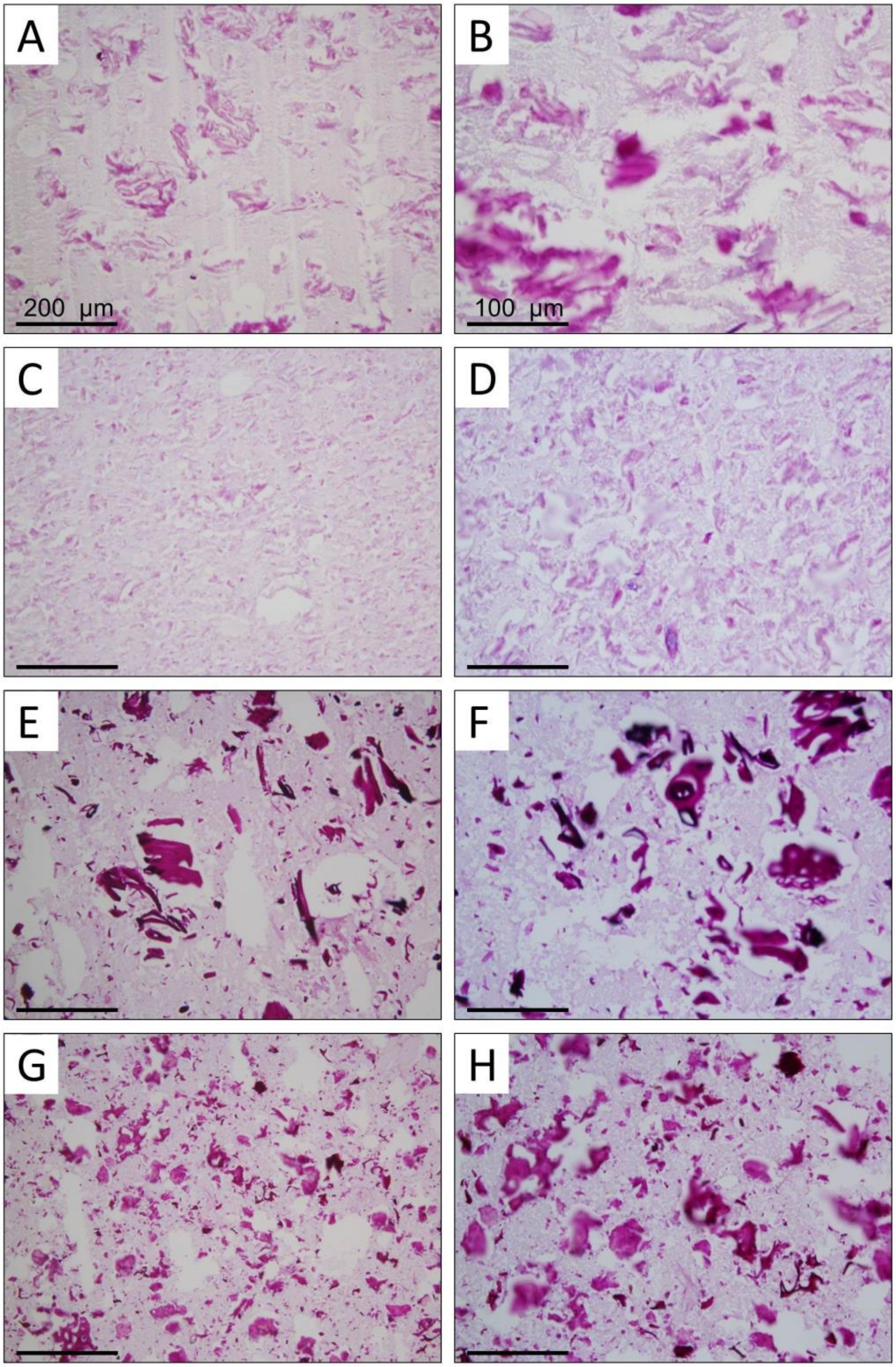

Fig. S3 Light micrographs of composite meat protein gels containing crystalline particles as fillers (mass fraction filler, m_f = 0.10). Panels (A,C,E,G) were acquired using a 10x objective, and those in Panels (B,D,F,H) were acquired with a 20x objective. The filler particles incorporated were MCC-102 (A,B), MCC-105 (C,D), oat fiber (E,F), and walnut flour (G,H).

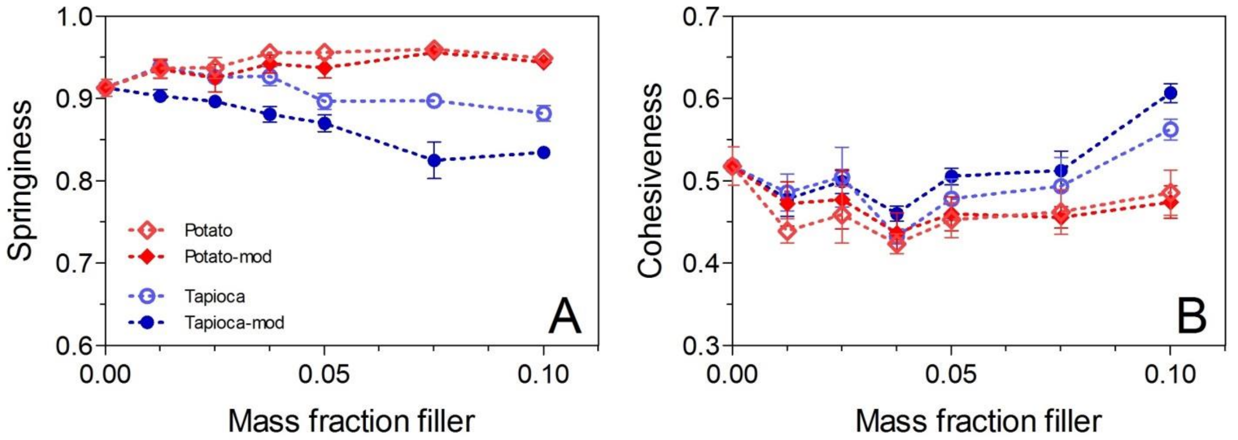

Fig. S4 Texture profile analysis (TPA) Springiness (A) and Cohesiveness (B) of comminuted meat protein gels containing various starches as fillers. Results are presented as a function of mass fraction filler.

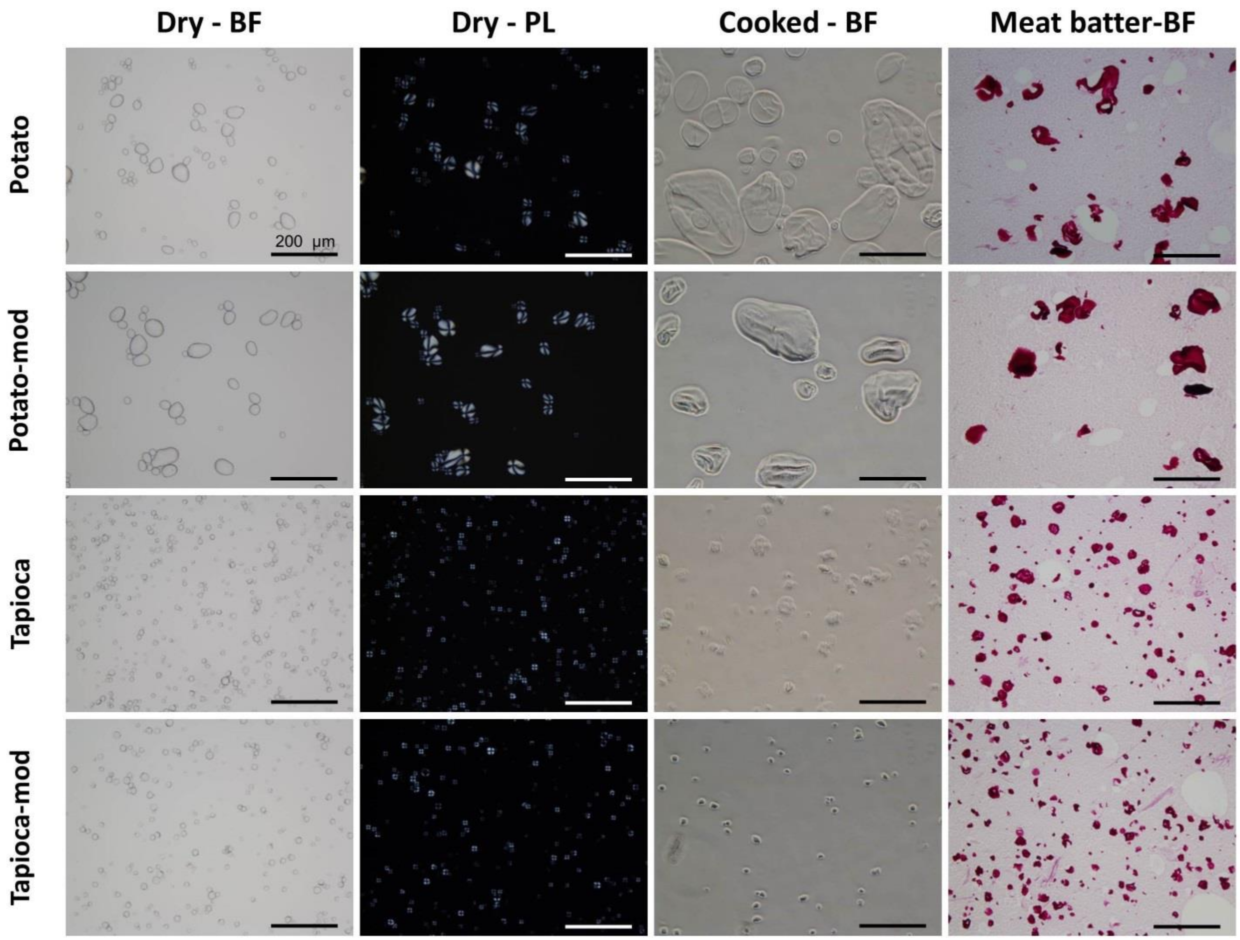

Fig. S5 Micrographs of starch particles used as fillers in comminuted meat batters, before and after thermal gelation (denoted "Dry" and "Cooked", respectively). BF indicates brightfield, and PL indicates polarized light. All images were acquired with a 10x objective. Far right column depicts particles dispersed in cooked meat batters (m_f=0.0125), as no crystallinity was observed in the Cooked starch particles in water.
